# Editorial: Systemic cellular immune responses and immunological biomarkers in emerging and re-emerging viral infections: an evolving landscape

**DOI:** 10.3389/fimmu.2024.1382350

**Published:** 2024-02-23

**Authors:** Iole Macchia, Valentina La Sorsa, Sonia Moretti

**Affiliations:** ^1^ Department of Oncology and Molecular Medicine, Istituto Superiore di Sanità, Rome, Italy; ^2^ Research Coordination and Support Service, Cori, Istituto Superiore di Sanità, Rome, Italy; ^3^ National HIV/AIDS Research Center (CNAIDS), Istituto Superiore di Sanità, Rome, Italy

**Keywords:** emerging diseases, immune response, viral infection, circulating biomarkers, highthroughput methods

Emerging/re-emerging infectious diseases (EIDs/REIDs) account for a significant proportion of outbreaks still impacting public health. EIDs represent recently recognized distinct infectious diseases, while REIDs are previously recognized infections that may re-emerge and rapidly spread, as to incidence or geographic range (1).

Most EIDs and REIDs are zoonotic and caused by RNA-viral pathogens that are highly mutable and can rapidly adapt to evade immune defence (2, 3). These include Avian influenza, Ebola and Marburg hemorrhagic- fevers, Lassa-fever, Dengue-fever, Yellow-fever, West Nile-fever, Zika, Swine-flu, severe acute respiratory syndrome (SARS), Middle-East respiratory syndrome (MERS), Chikungunya (vector-borne) and Coronavirus disease 2019 (COVID-19), the recent pandemic caused by SARS-CoV-2 coronavirus (4).

The balance between viral replication and host immunity dictates the outcome and severity of most viral diseases and T cell memory response is crucial for limiting or preventing viral reinfection, highlighting the rationale for host-directed intervention strategies providing long-term immune protection.

Several immunological blood biomarkers have been identified as key players in the monitoring, diagnosis and follow-up of various infectious diseases, thanks to new “Omic” technologies and high-throughput methods based on multiparametric flow cytometry (MFC).

The original Research articles and Reviews, collected in the present Research Topic, provide an overview and novel insights into the study of systemic innate and adaptive immune responses in emerging diseases.

A common feature in the pathogenesis of several EIDs is the uncontrolled inflammatory cytokine storm as the main driver of tissue damage, leading to immune-mediated multi-organ injury and disease progression. The mini-review of Agrati et al. addressed the role of immune response to emerging viral infections in immunocompromised hosts, at increased risk of morbidity and mortality. In this context, immunosuppressive conditions could be harmful in the initial phase of infection, when the host immune response is necessary to inhibit viral replication, but may paradoxically have beneficial effects in the later phase of disease, dampening the excessive inflammatory response and reducing the immune-mediated damage.

In their study, Soni et al. provided an in-depth analysis of T cell responses against the immunodominant SARS-CoV-2 spike, nucleocapsid and membrane proteins and the corresponding antigens from endemic non-SARS human coronaviruses (hCoVs), pathogens of common cold. Their findings highlight a pre-existing T-cell memory in subjects recovered from COVID-19 or vaccinated, retaining cross-reactivity between ancestral α- and β- hCoVs and multiple SARS-CoV-2 mutants. Based on the cross-reactivity patterns, authors suggested a potential universal T cell-based vaccination strategy, targeting multiple coronaviruses and established an *in-vitro* expansion protocol of universal anti-hCoV T-cells for adoptive immunotherapy.

Among the post-acute sequelae of COVID-19 (PASC), pulmonary fibrosis (PF) represents the most significant long-term respiratory morbidity. To ascertain the factors influencing PF progression for patients’ management and treatment, Bingham et al. evaluated immune mechanisms associated with recovery on peripheral blood mononuclear cells (PBMCs) from early- (ER) and late-resolving (LR) COVID-PF patients versus persistent PF. Using multiplex immunostaining they found a significantly lower relative abundance of circulating monocytes in LR compared to ER COVID-PF. Expression analyses obtained by scRNAseq single-cell transcriptomics, indicated that alarmins were the most significantly upregulated genes within LR COVID-PF circulating monocytes, while MHC class II molecules were downregulated in LR COVID-PF monocytes compared to ER COVID-PF. Conversely, CD8+ T effector cells showed an increased HLA-DR protein expression and gene upregulation in LR COVID-PF compared to ER COVID-PF, suggestive of prolonged immune activation.

Yoon et al. studied the immune dysregulation in PASC. They characterized the transcriptomic profiles of PBMCs from individuals who developed pulmonary PASC (PPASC) after SARS-CoV-2 infection, by droplet-based scRNA‐seq. Observations demonstrated that levels of myeloid-lineage cells (MLCs), monocytes and dendritic cells, were increased in PPASC compared to controls. MLCs from PPASC displayed up-regulation of PF-associated genes, while glycolysis metabolism-related genes were downregulated, suggesting that increased MLC proportions and the altered gene signatures may contribute to PPASC development.

Lesteberg et al. debated the role and responses of specific T-cell subsets in severe intensive care unit (ICU) and non-severe hospitalized COVID-19 patients, including those with chronic illnesses (diabetes and hypertension). They focused on T-cell expression of CD62L, a ligand for T-cell trafficking to lymph nodes and non-lymphoid tissues, including lung tissue during respiratory infections. They observed greater frequencies of CD62L+CD4+ T cells in severe compared to non-severe COVID-19 patients, and higher perforin+CD8+ T cells compared to recovered patients. Moreover, severe COVID-19 patients with diabetes and hypertension showed greater frequencies of T cells expressing CD62L, suggesting its role as prognostic marker.

Nucleated red blood cells (nRBC) are precursors, absent from peripheral blood under physiological conditions but associated with adverse outcomes in critically ill patients. SARS-CoV-2 infection may affect the bone marrow, and viral-induced cytokine storm and hypoxemia can engage mechanisms triggering nRBC release. Schmidt et al. assessed the predictive value of nRBC on mortality in a retrospective cohort of ICU patients with COVID-19 acute respiratory distress syndrome (ARDS). Results demonstrated that maximum nRBC count could predict disease severity and that ICU mortality risk increases incrementally with maximum nRBC count. Analyzing clinical parameters before and after the nRBC peak, non-survivors showed persistent inflammation, indicated by numerically persistent IL-6 levels. Maximum nRBC counts indicate disease severity among COVID-19 ARDS patients and, when compared to established clinical scoring systems, they should be considered as a late biomarker for ICU mortality.

The review by Macchia et al. discuss the role and potential utility of eosinophils as prognostic/predictive blood-based immune biomarkers in emerging respiratory viral diseases including COVID-19. These granulocytes, frequently associated with asthma exacerbation, may participate to antiviral response against respiratory viruses through the production of soluble mediators, such as cationic proteins, RNases and reactive nitrogen species. Indeed, eosinopenia is an indicator of severity among patients with COVID-19. Finally, this review highlights some relevant methodologies employed for the isolation and immunophenotyping of eosinophils.

As an approach to characterize host responses after infection or vaccination, Quach et al. determined gene expression signatures in circulating leukocytes of healthy donors, who received both seasonal influenza and smallpox vaccines. Gene expression profiles were separately analyzed in PBMCs, monocytes, B cells, and CD8 + T-cells isolated *ex vivo* from the peripheral blood and *in vitro* stimulated with either influenza virus or vaccinia virus. The majority of differentially expressed genes (DEGs) identified in PBMCs were also found in monocytes after either viral stimulation. No shared DEGs, were identified in infected CD8+ T cells whereas in B cells shared DEGs were expressed similarly after both virus stimulation. Gene set enrichment analysis demonstrated that shared DEGs were over-represented in innate signaling pathways, especially in monocytes, providing insights into virus-host interactions and specific targets for the development of novel virus-specific therapeutics.

We hope this Research Topic will provide interesting insights for future work exploring vaccine and therapeutic strategies against emerging and re-emerging infectious diseases.

## Author contributions

IM: Writing – original draft, Writing – review & editing. VS: Writing – original draft, Writing – review & editing. SM: Writing – original draft, Writing – review & editing.
